# Validation of an instrument to evaluate quality of life in the aging population: WHOQOL-AGE

**DOI:** 10.1186/1477-7525-11-177

**Published:** 2013-10-23

**Authors:** Francisco Félix Caballero, Marta Miret, Mick Power, Somnath Chatterji, Beata Tobiasz-Adamczyk, Seppo Koskinen, Matilde Leonardi, Beatriz Olaya, Josep Maria Haro, José Luis Ayuso-Mateos

**Affiliations:** 1 Department of Psychiatry, Universidad Autónoma de Madrid, Madrid, Spain; 2 Instituto de Salud Carlos III, Centro de Investigación Biomédica en Red de Salud Mental CIBERSAM, Spain; 3 Department of Psychiatry, Hospital Universitario de La Princesa, Instituto de Investigación Sanitaria Princesa (IP), Madrid, Spain; 4 Section of Clinical and Health Psychology, University of Edinburgh, Edinburgh, UK; 5 Department of Health Statistics and Information Systems, World Health Organization, Geneva, Switzerland; 6 Department of Medical Sociology, Jagiellonian University Medical College, Krakow, Poland; 7 National Institute for Health and Welfare, Helsinki, Finland; 8 Fondazione IRCCS, Neurological Institute Carlo Besta, Milano, Italy; 9 Parc Sanitari Sant Joan de Déu, Universitat de Barcelona, Sant Boi de Llobregat, Barcelona, Spain

## Abstract

**Background:**

There is a need for short, specific instruments that assess quality of life (QOL) adequately in the older adult population. The aims of the present study were to obtain evidence on the validity of the inferences that could be drawn from an instrument to measure QOL in the aging population (people 50+ years old), and to test its psychometric properties.

**Methods:**

The instrument, WHOQOL-AGE, comprised 13 positive items, assessed on a five-point rating scale, and was administered to nationally representative samples (n = 9987) from Finland, Poland, and Spain. Cronbach’s alpha was employed to assess internal consistency reliability, whereas the validity of the questionnaire was assessed by means of factor analysis, graded response model, Pearson’s correlation coefficient and unpaired *t*-test. Normative values were calculated across countries and for different age groups.

**Results:**

The satisfactory goodness-of-fit indices confirmed that the factorial structure of WHOQOL-AGE comprises two first-order factors. Cronbach’s alpha was 0.88 for factor 1, and 0.84 for factor 2. Evidence supporting a global score was found with a second-order factor model, according to the goodness-of-fit indices: CFI = 0.93, TLI = 0.91, RMSEA = 0.073. Convergent validity was estimated at *r* = 0.75 and adequate discriminant validity was also found. Significant differences were found between healthy individuals (74.19 ± 13.21) and individuals with at least one chronic condition (64.29 ± 16.29), supporting adequate known-groups validity.

**Conclusions:**

WHOQOL-AGE has shown good psychometric properties in Finland, Poland, and Spain. Therefore, considerable support is provided to using the WHOQOL-AGE to measure QOL in older adults in these countries, and to compare the QOL of older and younger adults.

## Background

The World Health Organization Quality of Life Assessment (WHOQOL) is an instrument to measure quality of life (QOL). It has been simultaneously developed in different cultures and languages in order to make it applicable across cultures [1]. There are some areas of QOL that may be more relevant for older adults; therefore, specific instruments that assess QOL adequately in the older adult population are needed [2]. The present study aimed to validate an instrument, the WHOQOL-AGE, built upon previous WHOQOL instruments, which is relatively short to use, e.g., in large-scale population studies or in busy clinical settings; use this instrument to measure QOL in an aging population; and test its psychometric properties in terms of its validity and reliability.

Several versions of the WHOQOL instruments have been shown to have good psychometric properties in terms of reliability, validity and sensitivity to change in different population groups. WHOQOL-100 is a reliable and valid measure of QOL for use in a diverse range of cultures [1] which consists of 24 facets grouped into six domains, whereas WHOQOL-BREF is a reduced 26-item version comprising four domains: physical, psychological, social and environment [3]. The EUROHIS-QOL eight-item index [4] is a brief questionnaire based on WHOQOL-100 and WHOQOL-BREF. It has shown good cross-cultural performance in ten European countries, as well as satisfactory convergent and discriminant validity.

In order to understand the QOL of older adults, some instruments to measure QOL in the elderly, such as the Elderly Quality of Life Index (EQOLI) [5] and the Quality of Life Scale for Elderly (QOLS-E) [6], have been developed. EQOLI was developed in Brazil to monitor longitudinal change in QOL, as well as to evaluate the impact on QOL of behavior, intervention, and treatment. The instrument comprises eight domains and 43 items [7]. The QOLS-E was developed and validated in a sample of the institutionalized population in Japan, and showed an adequate factor structure, although its reliability was not very high [6].

The WHOQOL-OLD is a supplementary module for the WHOQOL for use with older adults, developed using the WHOQOL methodology, in which a simultaneous approach to instrument development is employed in different cultures [2]. Recently, short versions of WHOQOL-OLD have also been developed [8]. Since WHOQOL-OLD needs to be administered together with WHOQOL-BREF, its administration, even when using the short versions of WHOQOL-OLD, requires a long time. Consequently, there is still a need to identify a parsimonious set of items to evaluate QOL in older adults in the general population that can be administered when time is at a premium, e.g. in population-based or clinical studies when other additional data need to be collected, depending on the primary purpose of the study. WHOQOL-AGE is attempting to cover this need, since it is a short instrument, designed to be administered in general population studies, which covers the areas of QOL that are specific to older adults.

WHOQOL-AGE has been designed specifically for the aging population, but in order to understand the transition of aging, it is also important to be able to compare the QOL of the aging population with younger people. The validation process of WHOQOL-AGE will, therefore, be carried out in the aging population and in the population aged 18–49 years, in order to make sure that the instrument also allows comparisons with younger populations.

## Methods

### Design and procedure

The “Collaborative Research on Ageing in Europe (COURAGE in Europe)” is an observational, cross-sectional study of the general non-institutionalized adult population reached though household interviews. The sample is representative of three European countries (Finland, Poland, and Spain), which were selected to give a broad representation across different geographical European regions, taking into consideration their population and health characteristics.

Face-to-face interviews using Computer-Assisted Personal Interviewing (CAPI) were carried out at the respondents’ homes. All of the interviewers participated in a training course for the administration of the survey. A total of 18 trainers from the three countries (six from Finland, eight from Poland, and four from Spain) attended a central five-day training in English, and they then trained the local interviewers of each country in the local languages (Finnish, Polish, and Spanish). The number of interviewers in the local trainings ranged from 14 in Finland to 55 in Poland. The surveys were conducted in 2011–2012.

### Sample

A multi-stage clustered design was used to obtain nationally representative samples. A probability proportion to size design was used to select clusters. In Poland and Spain, an enumeration of existing households was carried out within each cluster to obtain an accurate measurement of size. In Finland, systematic sampling of individuals within each cluster was applied.

Initially, 10 800 respondents were recruited (1976 from Finland, 4071 from Poland, and 4753 from Spain). As in many other aging studies, such as SHARE [9], HRS [10], ELSA [11], TILDA [12], MHAS [13] or SAGE [14], people 50+ years old were evaluated in order to understand the transition of aging. Furthermore a group of subjects who were 18–49 was also included in order to make comparisons between younger and older people. A split technique was used to divide the overall sample into two groups: developmental and validation. 70% (*n* = 7560) was randomly assigned to the developmental sample, and the remaining 30% (*n* = 3240) to the validation sample, considering a similar proportion of respondents by country in each sample. The developmental sample was used to analyze the factorial structure of the WHOQOL-AGE by means of exploratory factor analysis, whereas the validation sample was used to assess the reliability and validity of the scale, using confirmatory factor analysis techniques and item response theory methods. The individual response rate was 53.4% for Finland, 66.5% for Poland, and 69.9% for Spain.

If a participant was cognitively impaired and not able to respond to the interview, a proxy was asked some questions about the participant’s health. For the purposes of the present analyses, these participants were not included.

### Measures

Items from WHOQOL-AGE were derived as an adaptation from the EUROHIS-QOL eight-item index [4] and from the WHOQOL-OLD short form version 1, which comprises six items [8]. A pilot study was carried out in 2010 in the three countries, and based on the feedback from the interviewers and on the preliminary analyses, one question from WHOQOL-OLD was deleted (*How concerned are you about how your life will end?*) and some wording was changed. Thus, the new instrument, WHOQOL-AGE, comprises 13 positive items (eight derived from EUROHIS-QOL and five from WHOQOL-OLD), assessed on a five-point rating scale.

Furthermore, participants answered questions regarding their overall satisfaction with life, net affect, and presence of chronic conditions. These measures were used, respectively, to evaluate convergent, discriminant, and known-groups validity.

To evaluate overall satisfaction with life (SWL), respondents were asked: *Taking all things together, how satisfied are you with your life as a whole these days?*, ranking their answer on a scale ranging from 1 = *very dissatisfied*, to 5 = *very satisfied*.

Net affect was assessed with an abbreviated version of the Day Reconstruction Method [15], designed to be used in general population surveys. Participants reconstructed a portion of their previous day’s activities and responded to questions about each episode, including what they were doing and the extent to which they experienced various feelings on a scale ranging from 0 (*not at all*) to 6 (*very much*), with the remaining points unlabelled [16,17]. Individual net affect was calculated by averaging two positive emotions (calm/relaxed and enjoying) minus five negative ones (worried, rushed, irritated/angry, depressed, and tense/stressed), weighting by activity duration. Net affect scores ranged from -6 to 6, with higher scores representing a better affective state.

Participants were also asked questions concerning their sociodemographic characteristics, and the presence of five chronic conditions (depression, arthritis, angina, diabetes, and asthma) during the previous 12 months was assessed. Individuals were considered to have the condition when they had been diagnosed with the condition and had been taking medication or other treatment during the previous 12 months, or when they reported the presence of the core symptoms of the condition during the previous 12 months.

The questions that had not been previously translated and validated in the local languages were translated from English into Finnish, Polish, and Spanish, following the World Health Organization translation guidelines for assessment instruments, which included a forward translation, a targeted back-translation, review by a bilingual expert group, and a detailed report on the translation process. The study was approved by the Bioethical Committee, Jagiellonian University, Krakow, Poland; Ethics Review Committee, Parc Sanitari Sant Joan de Déu, Barcelona, Spain; Ethics Review Committee, La Princesa University Hospital, Madrid, Spain; and the Ethics Review Committee, National Public Health Institute, Helsinki, Finland. Written information consent from each participant was also obtained.

### Statistical analysis

Participants who did not complete the interview and did not respond to the QOL section were excluded, as were participants who responded to the QOL section but did not respond to one or more items of WHOQOL-AGE. Frequency analysis and descriptive statistics were used to analyze the demographic characteristics of the developmental and validation samples, after excluding missing values. Differences in proportions and scores between both samples were analyzed using Chi-square tests and unpaired *t*-tests.

#### Developmental sample

An Exploratory Factor Analysis (EFA) using a polychoric correlation matrix was conducted on the developmental sample to detect the latent structure among WHOQOL-AGE items. Velicer’s Minimum Average Partial (MAP) test [18] was employed to select the number of factors to extract. Geomin rotation for correlated factors was used and each item was associated with the factor in which it had the highest loading. The EFA was carried out separately on people less than and more than 50 years old. In order to assess the factorial equivalence between the two populations, the factor congruence coefficient [19] was calculated, which measures the degree of similarity between factor structures obtained in two independent samples. Interpretation of this coefficient is similar to the Pearson’s product moment correlation. A value of 0.90 is typically considered necessary to suggest factor congruence [20].

#### Validation sample

A Confirmatory Factor Analysis (CFA), using maximum likelihood estimation with robust standard errors (MLR estimation), was used to assess how well the data fit the theoretical model and therefore to confirm the factorial structure suggested by the EFA carried out on the developmental sample. Goodness-of-fit of the model was evaluated according to the standard recommendations [21,22]. Values of the Comparative Fit Index (CFI) and Tucker-Lewis Index (TLI) above 0.90 were considered to represent an adequate fit; values of Root Mean Square Error of Approximation (RMSEA) less than 0.08 indicated a good fit [23]. χ^2^ test of goodness-of-fit was not reported. Since the χ^2^ statistic is sensitive to sample size [24], the χ^2^ values might be inflated (statistically significant) due to the large size of the sample, which might erroneously imply a poor data-to-model fit [25]. Burnham and Anderson [26] noted that model goodness-of-fit based on statistical tests becomes irrelevant with large sample sizes. One common assumption in these models is that a parameter is equal to a given value, often zero (e.g. saying there is no direct relationship between two variables). Modification indices were employed to evaluate how reasonable these assumptions are, by observing what happens when these assumptions are relaxed.

Moreover, the goodness-of-fit of each model was assessed by means of the Bayesian Information Criterion (BIC) proposed by Schwarz [27], which is asymptotically consistent with large sample sizes. Information criteria are entropy-based measures of the goodness-of-fit of a statistical model. They can be applied to models with parameters estimated using maximum likelihood methods. In the case of factor analysis, the aim is to create a factor model that balances complexity (number of factors) with the amount of variance explained. The definition of the information criteria implies that a smaller value indicates a better model.

Since the item responses are polytomous and ordered, a Graded Response Model (GRM) was employed for each of the factors obtained [28]. In the GRM, the values of the discrimination parameter and the item information function were estimated for each item. The discrimination parameter represents the ability of an item to discriminate between people with different levels of an underlying trait; and the total information was calculated by adding up the item information values—the greater the value, the more contribution to the measure of the factor.

In order to find evidence for the use of a global score on the WHOQOL-AGE, a second-order confirmatory factor analysis was applied to test the accuracy of a model with a second-order factor comprising the first-order factors obtained previously by means of exploratory and confirmatory factor analyses. If there is only one second-order factor, then there must be at least three first-order factors if the model is to be identified [29]. To solve under-identification problems, the first-order factor variance was fixed to 1, and the mean and the variance of the second-order factor were fixed to 0 and 1, respectively.

The internal consistency reliability was assessed by means of Cronbach’s alpha. As suggested by Bland & Altman [30], a Cronbach’s alpha of 0.70 or higher was considered to indicate adequate reliability. Convergent validity was evaluated by the correlation between the SWL item and the global WHOQOL-AGE score. Discriminant validity was evaluated by means of Pearson’s correlation coefficient between the WHOQOL-AGE score and the net affect score. The method described by Raykov [31] was used to test whether the discriminant validity coefficient was sufficiently lower than the convergent validity coefficient, a condition posited by Campbell & Fiske [32] as evidence supporting construct validity. In order to assess the known-groups validity of the questionnaire, the WHOQOL-AGE score was compared for healthy and non-healthy populations. Participants were defined as healthy if they did not present any of the chronic conditions assessed (depression, arthritis, angina, asthma, and diabetes), whereas they were defined as non-healthy if they had at least one of those chronic conditions. Mean scores were compared by means of unpaired *t*-tests, and the magnitude of the difference was measured by Hedges’ *g* effect size coefficient. The results obtained in terms of reliability and validity for the WHOQOL-AGE were compared with those obtained using the EUROHIS-QOL, and the five items from the WHOQOL-OLD short form version 1 that were included in the WHOQOL-AGE questionnaire.

Normative values, including main percentiles of the distribution of WHOQOL-AGE scores, were calculated across countries and for different age groups: 18–29, 30–39, 40–49, 50–59, 60–69, 70–79, 80–89, and 90+ years. The data to obtain the normative values were weighted to account for the sampling design in order to generalize the results to the population in each country. Finally, the cumulative distribution of WHOQOL-AGE scores by country was presented across the population aged 18–49 years and the population aged 50 and over.

Analyses corresponding to GRM were carried out using the *ltm* package [33] in R [34]. Mplus version 6 [35] was employed for factor analysis modeling. The rest of the analyses were performed using Stata version 11 [36].

## Results

The final sample used comprised 9987 participants. Significant differences between the included and the excluded sample were found for age (58.10 ± 16.70 years for the included sample vs. 71.83 ± 16.29 years for the excluded sample, *t*(10 755) = -22.02, *p* < 0.001, Hedges’ *g* = 0.82), sex (56.7% females in the included sample vs. 64.7% females in the excluded sample, χ^2^(1) = 18.43, *p* < 0.001, Cramer’s *V* = 0.04), years of education (11.47 ± 5.17 vs. 8.62 ± 6.53, *t*(10 427) = 11.45, *p* < 0.001, Hedges’ *g* = 0.54), and marital status (60.3% married or in partnership in the included sample vs. 37.7% married or in partnership in the excluded sample, χ^2^(1) = 151.21, *p* < 0.001, Cramer’s *V* = 0.12); differences were not found regarding residential setting. Percentages by countries were, in the included population, 18.5% from Finland, 39.5% from Poland, and 42.1% from Spain; and in the excluded population, 16.1% from Finland, 16.1% from Poland, and 67.8% from Spain. Differences in association between country and included/excluded were significant (χ^2^(2) = 223.74, *p* < 0.001), although with moderate effect size (Cramer’s *V* = 0.14).

The sample was randomly split into a developmental sample (*n* = 6993) and a validation sample (*n* = 2994). In order to confirm that both subsamples were representative of the initial sample, the demographic characteristics of both populations were compared. Sociodemographic characteristics of these samples are shown in Table 1. Significant differences were not found between the developmental and the validation samples regarding the main sociodemographic characteristics.

**Table 1:** Sociodemographic characteristics and total EUROHIS-QOL scores in the developmental and validation samples

**Variable**	**Sample ( *n *)**		***t*/Chi-square**	***p*-value**
	**Developmental (6993)**	**Validation (2994)**		
≥ 50 years: *n* (%)	5285 (75.6)	2280 (76.2)	0.38	0.54
Female: *n* (%)	3978 (56.9)	1688 (56.4)	0.22	0.64
Country				
Finland	1260 (18.0)	585 (19.5)	3.70	0.16
Poland	2760 (39.5)	1180 (39.4)		
Spain	2973 (42.5)	1229 (41.0)		
Rural setting: *n* (%)	1879 (26.9)	789 (26.4)	0.29	0.59
Currently employed: *n* (%)	2521 (36.1)	1045 (34.9)	1.20	0.27
Married or in partnership: *n* (%)	4252 (60.8)	1771 (59.2)	2.39	0.12
Years of education, mean ± S.D.	11.47 ± 5.11	11.48 ± 5.29	-0.05	0.96
EUROHIS-QOL score, mean ± S.D.	71.20 ± 15.52	70.77 ± 15.78	1.24	0.22

Unpaired *t* test for years of education and EUROHIS-QOL score; Chi-square test for the other variables.

### Exploratory factor analysis (EFA)

In the developmental sample, the Velicer MAP criterion achieved the minimum value for the solution comprising two factors. The two-factor solution explained 62.8% of the total variance for people aged 18–49 years old, and 65% of the total variance for those aged 50+. In Table 2, factor loading estimates after Geomin rotation are shown for both age groups. Items Q2-Q8 loaded on the first factor, whereas items Q9-Q13 loaded on the second factor. Item Q1 presented a similar loading on the first and the second factors, and was considered as belonging to both factors. A similar factor structure was found between both samples, with Tucker’s congruence coefficient being 0.98 for the first factor and 0.96 for the second factor.

**Table 2:** Two-factor solution corresponding to EFA conducted on the developmental sample (n = 6993): factor loading estimates after Geomin rotation

	**People 18–49 years old (*n*** **= 1708)**		**People 50+ years old (*n*** **= 5285)**	
	**Factor 1 loadings (s.e.)**	**Factor 2 loadings (s.e.)**	**Factor 1 loadings (s.e.)**	**Factor 2 loadings (s.e.)**
*Q1 How would you rate your quality of life?*	**0.284 (0.031)**	**0.358 (0.031)**	**0.354 (0.018)**	**0.319 (0.019)**
**Q2. How satisfied are you with your hearing, vision or other senses overall?**	**0.766 (0.025)**	-0.065 (0.032)	**0.570 (0.017)**	0.138 (0.020)
*Q3. How satisfied are you with your health?*	**0.911 (0.021)**	-0.095 (0.028)	**0.732 (0.016)**	0.164 (0.020)
*Q4. How satisfied are you with yourself?*	**0.833 (0.021)**	0.065 (0.028)	**0.880 (0.011)**	-0.016 (0.015)
*Q5. How satisfied are you with your ability to perform your daily living activities?*	**0.894 (0.007)**	-0.002 (0.002)	**0.724 (0.014)**	0.210 (0.018)
*Q6. How satisfied are you with your personal relationships?*	**0.758 (0.025)**	0.100 (0.032)	**0.869 (0.012)**	-0.028 (0.017)
*Q7. How satisfied are you with the conditions of your living place (your home)?*	**0.563 (0.029)**	0.135 (0.035)	**0.639 (0.010)**	-0.005 (0.008)
**Q8. How satisfied are you with the way you use your time?**	**0.617 (0.028)**	0.149 (0.034)	**0.750 (0.013)**	0.043 (0.017)
*Q9. Do you have enough energy for everyday life?*	0.209 (0.027)	**0.645 (0.025)**	0.088 (0.014)	**0.809 (0.011)**
**Q10. How much control do you have over the things you like to do?**	-0.009 (0.006)	**0.868 (0.012)**	-0.066 (0.015)	**0.956 (0.011)**
**Q11. To what extent are you satisfied with your opportunities to continue achieving in life?**	0.055 (0.029)	**0.785 (0.025)**	0.001 (0.001)	**0.869 (0.004)**
*Q12. Do you have enough money to meet your needs?*	-0.015 (0.031)	**0.587 (0.029)**	0.028 (0.017)	**0.589 (0.015)**
**Q13. How satisfied are you with your intimate relationships in your life?**	0.247 (0.033)	**0.383 (0.033)**	0.228 (0.017)	**0.436 (0.017)**

**In bold**, items from WHOQOL-OLD short form version 1, *in italics*, items from EUROHIS-QOL. All the response options use a five-point rating scale, ranging from *very bad* to *very good* for Q1, from *very dissatisfied* to *very satisfied* for Q2 to Q8, from *not at all* to *completely* for Q9 to Q12, and from *not at all* to *an extreme amount* for Q13.

### Confirmatory factor analysis (CFA)

A CFA was carried out on the validation sample in order to assess the suitability of the factor model proposed, comprising two correlated factors. Due to the similar factor structure found in the EFA in both age groups, the CFA was conducted over the pooled sample. One of the two factors loaded on items Q1, Q2, Q3, Q4, Q5, Q6, Q7, and Q8, and the other one loaded on items Q1, Q9, Q10, Q11, Q12, and Q13. Goodness-of-fit indices associated with the two-factor solution were: CFI = 0.90, TLI = 0.87, RMSEA = 0.085 [90% CI = (0.081, 0.089)], BIC = 82176.77. On the other hand, an alternative comprising only one factor showed a poor fit: CFI = 0.81, TLI = 0.78, RMSEA = 0.113 [90% CI = (0.110, 0.117)], BIC = 83890.45. According to model modification indices, the fit for the two-factor solution was improved by allowing error covariance between items Q3 and Q5 to covary with the first factor (revised model: CFI = 0.92, TLI = 0.90, RMSEA = 0.077 [90% CI = (0.073, 0.080)], BIC = 81794.16). This improvement in fit demonstrated that the two-factor model fitted the data, but it also showed the strong relationship between satisfaction with health and satisfaction with the ability to perform daily living activities. The standardized factor loadings for all items were positive and significant, ranging from 0.35 to 0.83 for the first factor, and from 0.30 to 0.83 for the second one. Correlation between factors was 0.75 [95% CI = (0.71, 0.79)].

### Graded response model (GRM)

Graded Response Models were carried out on each factor in the validation sample, considering item Q1 as belonging to both factors. Table 3 shows discrimination parameters and the total information explained by the items in each factor. By means of the Test Information Curve, it was observed that the five items added to the eight-item EUROHIS-QOL provided 20.81% of the total information corresponding to factor 1, and 58.72% of the information corresponding to factor 2 (taking into account that items Q2 and Q8 loaded on factor 1; and items Q10, Q11, and Q13, on factor 2).

**Table 3:** Results of the WHOQOL-AGE scale analysis based on the graded response model for each factor (n = 2994)

**Items**	**Factor 1**		**Factor 2**	
	**Discrimination parameter**	**Total information explained (%)**	**Discrimination parameter**	**Total information explained (%)**
**Q1**	1.544	7.95	1.398	9.71
**Q2**	1.977	9.66	-	-
**Q3**	2.704	14.51	-	-
**Q4**	3.311	19.11	-	-
**Q5**	3.153	16.91	-	-
**Q6**	2.635	14.24	-	-
**Q7**	1.392	6.47	-	-
**Q8**	2.136	11.15	-	-
**Q9**	-	-	3.190	23.85
**Q10**	-	-	3.392	26.09
**Q11**	-	-	3.213	24.41
**Q12**	-	-	1.369	7.71
**Q13**	-	-	1.377	8.22

**Figure 1:**
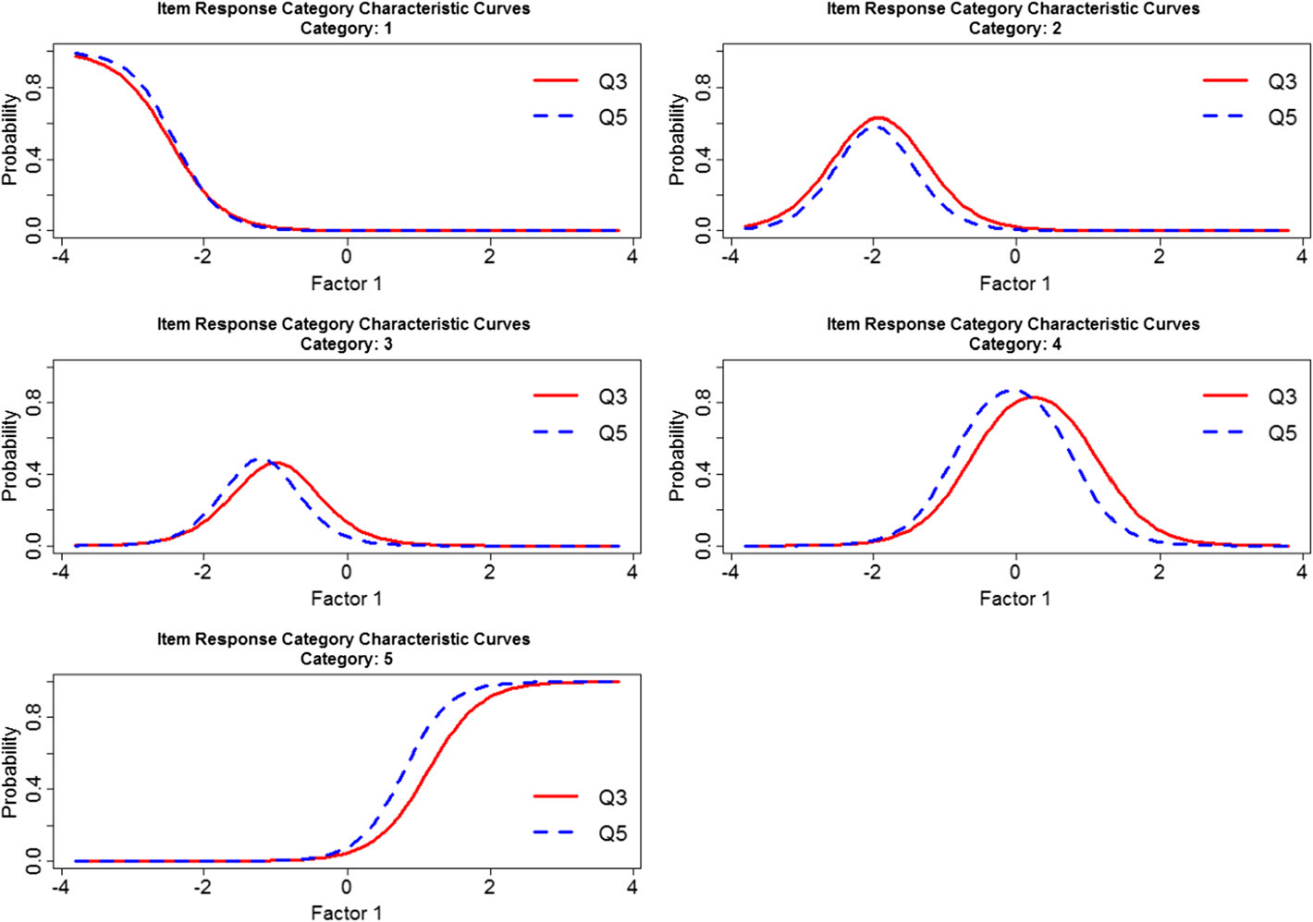
Item Response Category Characteristic Curves associated with items Q3 and Q5.

**Figure 2:**
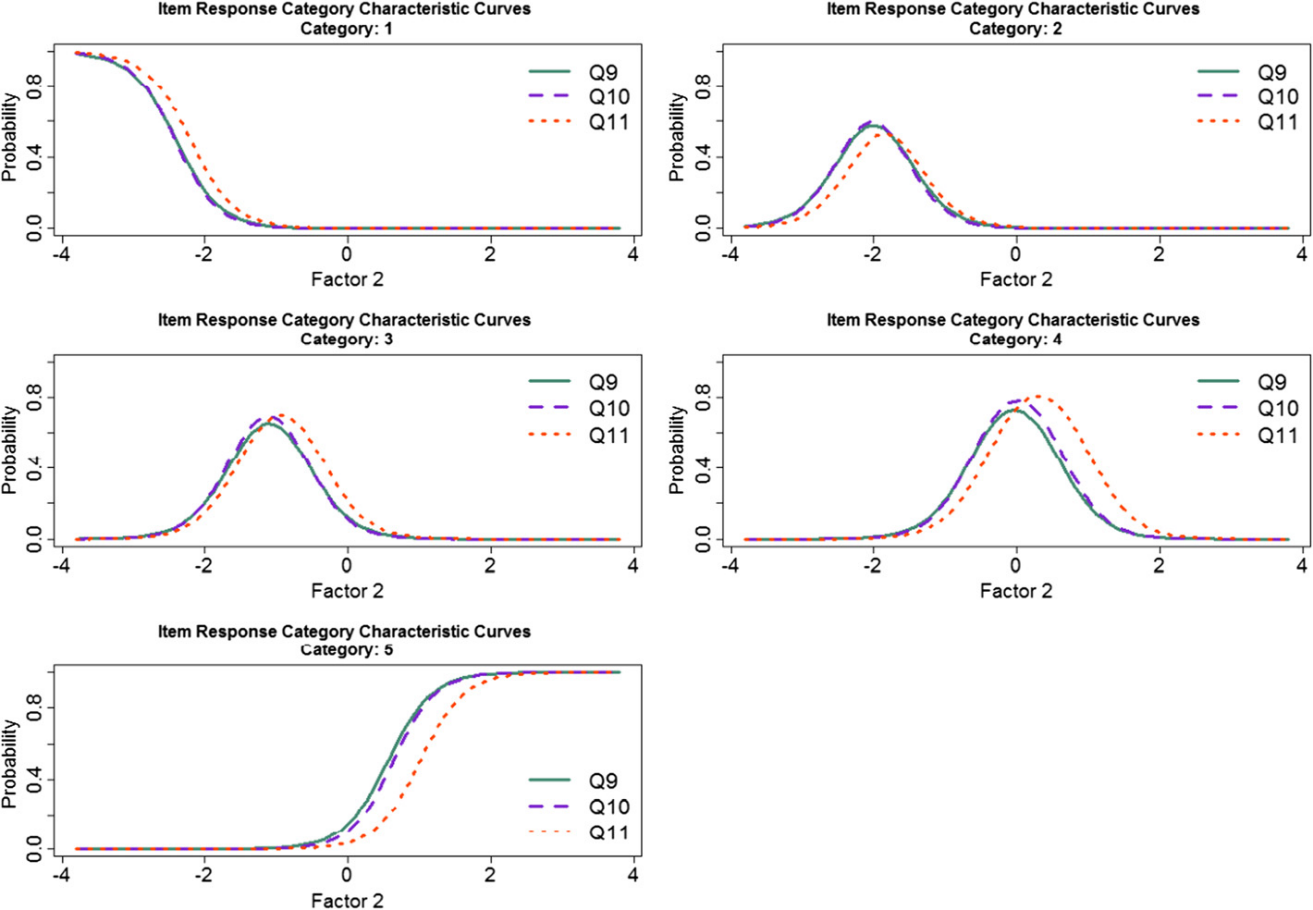
Item Response Category Characteristic Curves associated with items Q9, Q10, and Q11.

A useful comparison between items can be performed by plotting the Item Characteristic Curves for each category separately. Items Q3 and Q5 were highly related, with a correlation coefficient equal to 0.81. According to the Item Response Category Characteristic Curves (Figure 1), these items had a very similar effect on the construct corresponding to the first factor. Moreover, items Q9, Q10 and Q11 had a similar effect on the construct corresponding to the second factor (Figure 2). Mean polychoric correlation between these three items was 0.76, with correlation coefficients among these three items ranging from 0.72 to 0.78.

These analyses were also carried out separately in both age groups (18–49 and 50+ years), and very similar results were found. For example, in the 50+ age group, the five items added to the eight-item EUROHIS-QOL provided 27.87% of the total information corresponding to factor 1, and 58.55% of the information corresponding to factor 2. These percentages were 24.15% and 60.81%, respectively, in the 18–49 age group.

### Scoring WHOQOL-AGE

Considering the solution comprising two factors, a score for each factor was obtained. Items with a similar performance (according to the Item Response Category Characteristic Curves) and a strong relationship between them, were combined in the scoring method proposed. Taking into account the similar performance of items Q3 and Q5; of items Q9, Q10 and Q11; and the similar loading of item Q1 on both factors, the following formula was proposed to calculate a score for each factor:

(1) $$F1=\frac{Q1}{2}+Q2+\frac{Q3+Q5}{2}+Q4+Q6+Q7+Q8$$

(2) $$F2=\frac{Q1}{2}+\frac{Q9+Q10+Q11}{3}+Q12+Q13$$

Finally, to find support to obtain a global score on WHOQOL-AGE based on the scores obtained for factor 1 and factor 2, a second-order confirmatory factor analysis was constructed over the first-order factors *F1* and *F2*. Adequate goodness-of-fit was found for this model: CFI = 0.93, TLI = 0.91, RMSEA = 0.073 [90% CI = (0.069, 0.077)], BIC = 81593.19. Scores obtained in equations (1) and (2) were transformed to the percentile scale, and then the global score on WHOQOL-AGE was defined as the average of these scores.

### Reliability and validity

Adequate Cronbach’s alpha values were found for each of the two latent factors (α = 0.88 for factor 1, α = 0.84 for factor 2, α = 0.91 for the entire scale). When analyses were performed separately for each country, Cronbach’s alpha values were 0.82 in Finland, and 0.89 in Poland and Spain for factor 1; and 0.77 in Finland, and 0.84 in Poland and Spain for factor 2. For the entire scale, Cronbach’s alpha values were 0.87 in Finland, and 0.91 in Poland and Spain.

Mean inter-item correlation for the WHOQOL-AGE items in the pooled sample was 0.44 for factor 1, 0.47 for factor 2, and 0.45 for the entire scale. In the case of EUROHIS-QOL and the five items from the WHOQOL-OLD short form version 1, Cronbach’s alpha values for the pooled sample were 0.86 and 0.79, respectively. Mean inter-item correlation was 0.46 for the EUROHIS-QOL items and 0.39 for the WHOQOL-OLD short version items.

The convergent validity of the WHOQOL-AGE was estimated at 0.75 [95% CI = (0.73, 0.77)]. Regarding discriminant validity, a moderate correlation was found between WHOQOL-AGE and net affect [*r* = 0.35; 95% CI = (0.31, 0.38)]. The resulting 95% CI for the difference between these convergent and discriminant validity coefficients was (0.37, 0.44). This result suggests, with high confidence, that the convergent validity coefficient considered was markedly higher in the population than the discriminant validity coefficient. Similar values for correlation coefficients were found across countries (results available from the authors upon request), with the only exception being the correlation between WHOQOL-AGE score and net affect, which was lower in Finland [*r* = 0.21, 95% CI = (0.17, 0.26)].

In Table 4, these reliability and validity coefficients are shown separately by age groups: 18–49 and 50+ years. These coefficients are reported for the WHOQOL-AGE, the EUROHIS-QOL, and the five items from the WHOQOL-OLD short form version 1. In the case of the WHOQOL-AGE, the Cronbach’s alpha values were α = 0.89 for factor 1 and α = 0.85 for factor 2 in the older population; these values were, respectively, 0.86 and 0.80 in the 18–49 age group. The five items from the WHOQOL-OLD short form version 1 that were included in the WHOQOL-AGE questionnaire showed lower reliability in the 18–49 age group, whereas the Cronbach’s alpha values for the WHOQOL-AGE were very similar, although slightly higher than for the EUROHIS-QOL, in the older population. Moreover, significant differences were found between healthy individuals (*n* = 1795) and individuals having at least one chronic condition (*n* = 1199), with higher scores on WHOQOL-AGE for healthy people (74.19 ± 13.21 vs. 64.29 ± 16.29, *t* (2992) = 18.30, *p* < 0.001). The effect size associated with this difference was considerable (Hedges’ *g* = 0.64). Significant differences were also found in the analysis carried out separately by countries, with effect sizes ranging from 0.54 to 0.79. These results suggested adequate known-groups validity.

**Table 4:** Reliability and validity coefficients for WHOQOL-AGE, EUROHIS-QOL and WHOQOL-OLD short form version 1, in the 18–49 and 50+ age groups

	**WHOQOL-AGE**	**EUROHIS-QOL**	**WHOQOL-OLD**
*18-49 years*			
Cronbach’s alpha	0.89	0.85	0.72
Mean inter-item correlation	0.39	0.41	0.34
Convergent validity (CV)	0.73 (0.70, 0.76)	0.74 (0.71, 0.77)	0.63 (0.58, 0.67)
Discriminant validity (DV)	0.35 (0.29, 0.41)	0.34 (0.28, 0.40)	0.36 (0.29, 0.42)
95% CI CV-DV	(0.29, 0.47)	(0.31, 0.49)	(0.19, 0.36)
*50+ years*			
Cronbach’s alpha	0.92	0.87	0.80
Mean inter-item correlation	0.46	0.46	0.44
Convergent validity (CV)	0.75 (0.73, 0.77)	0.76 (0.74, 0.79)	0.69 (0.67, 0.71)
Discriminant validity (DV)	0.37 (0.33, 0.40)	0.36 (0.33, 0.40)	0.35 (0.31, 0.39)
95% CI CV-DV	(0.34, 0.42)	(0.37, 0.45)	(0.29, 0.37)

CV = Pearson’s correlation coefficient (95% CI) with the Satisfaction With Life (SWL) item; DV = Pearson’s correlation coefficient (95% CI) with the net affect score; 95% CI CV-DV = 95% confidence interval for the difference between the convergent and the discriminant validity coefficients.

**Figure 3:**
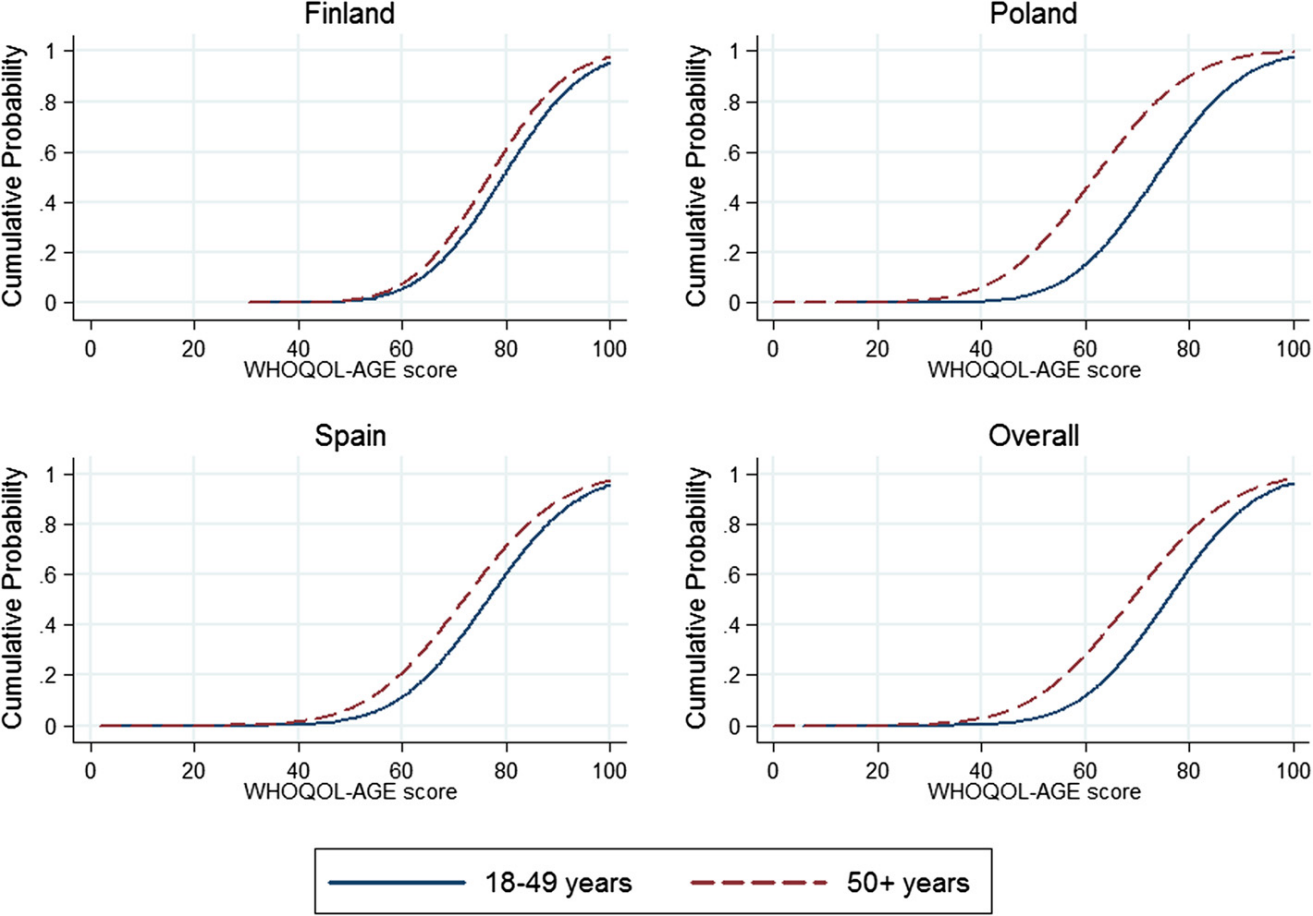
Smoothed Gaussian cumulative distribution functions of the WHOQOL-AGE scores across the population aged 18–49 years and the population aged 50 and over.

In terms of score distributions, the observed range was similar to the theoretical range (from 0 to 100), indicating that the measure covers the full range of the QOL continuum, although the distribution had negative skew (Fisher-Pearson coefficient of skewness = -0.69), indicating that most of the people reported a good QOL, as expected, given that the study sample came from the general population and not from clinical settings. Floor effects were negligible (there was only one person with a score of zero, the worst QOL), and ceiling effect was also acceptable (1.4%). Scores on WHOQOL-AGE decreased as age increased, as can be seen in the table of normative values (see Table 5). The cumulative distribution of WHOQOL-AGE scores for the 18–49 and 50+ age groups can be observed in the Figure 3, supporting results that suggest a lower QOL for the older population, according to their WHOQOL-AGE scores.

**Table 5:** Normative values: WHOQOL-AGE mean estimates, standard errors (s.e.) and estimated mean scores at the main percentiles, by age group, for Finland, Poland, and Spain

**Age group**	***n***	**Mean (s.e.)**	**5th**	**10th**	**25th**	**50th**	**75th**	**90th**	**95th**
**Finland**									
18-29	114	79.50 (1.35)	56.87	62.68	72.12	81.23	88.97	93.41	97.62
30-39	128	79.89 (1.28)	61.13	63.55	72.57	80.27	88.74	95.70	98.08
40-49	235	79.03 (0.82)	56.27	63.78	72.62	79.76	87.77	94.51	96.15
50-59	439	77.02 (0.52)	56.59	62.04	70.01	78.34	85.99	92.12	94.51
60-69	482	76.83 (0.60)	54.85	61.81	70.47	77.34	85.53	92.58	95.19
70-79	266	76.80 (0.72)	58.24	62.73	70.01	77.66	83.84	90.52	93.54
80-89	163	73.18 (0.85)	56.00	61.26	68.18	74.04	80.36	87.64	90.29
90+	18	69.10 (2.63)	39.70	60.03	64.84	70.56	75.00	83.70	83.70
**Poland**									
18-29	407	77.45 (0.93)	54.67	63.10	71.15	78.25	86.54	92.95	98.81
30-39	349	73.97 (1.11)	53.21	57.19	67.86	75.00	80.59	88.19	94.05
40-49	274	67.17 (1.12)	43.86	51.92	60.35	68.59	75.00	83.38	85.76
50-59	1050	65.32 (0.56)	41.67	47.39	57.14	67.35	74.54	80.27	83.70
60-69	861	63.44 (0.75)	39.33	45.42	55.08	65.20	72.57	78.34	83.88
70-79	527	59.53 (0.89)	35.99	42.45	50.00	61.54	69.28	75.00	79.12
80-89	435	54.03 (1.19)	26.10	35.44	46.34	55.95	65.48	72.89	77.38
90+	37	55.92 (2.99)	25.27	39.29	43.13	55.59	67.86	75.00	75.00
**Spain**									
18-29	247	79.46 (1.01)	55.68	63.46	72.62	79.53	87.77	97.25	100.00
30-39	296	77.39 (0.95)	56.18	60.58	69.87	77.43	87.27	92.86	97.25
40-49	372	73.97 (0.91)	48.35	57.69	67.72	75.00	83.70	91.76	95.33
50-59	1137	72.91 (0.68)	45.33	54.81	65.93	75.00	82.14	89.93	94.14
60-69	960	72.76 (0.72)	46.47	54.44	64.97	73.81	82.01	90.11	95.33
70-79	880	70.40 (0.84)	39.79	50.00	63.64	72.99	79.12	86.54	92.58
80-89	284	66.60 (1.15)	40.38	48.90	59.25	68.32	76.65	85.44	93.41
90+	26	66.03 (2.94)	37.64	43.73	56.23	71.43	75.73	78.34	78.34

Weighted data.

The age range of the samples was 18–97 years in Finland, 18–98 years in Poland, and 18–104 in Spain.

## Discussion

The present study aimed to validate an instrument to measure QOL in an aging population. WHOQOL-AGE has shown good psychometric properties in Finland, Poland, and Spain. Adequate goodness-of-fit indices were found according to the standard recommendations of Structural Equation Modeling literature [21-23]. These indices confirmed that the factorial structure of WHOQOL-AGE comprises two first-order factors, one loaded by items Q2 to Q8, and the other one loaded by items Q9 to Q13, with item Q1 loading on both factors. However, by means of a second-order confirmatory factor analysis, evidence was found supporting that these two factors belong to a more general construct. The similar factor structure in the population aged 18–49 years and in the population aged 50+, the results obtained in the pooled sample during the validation process, and the analyses carried out separately for both age groups, suggested that this instrument could be employed in the population aged 18–49 in order to compare their QOL with the older adults.

A score for each component and a global score for WHOQOL-AGE are proposed; this method would involve recombining some items before converting the score on each factor into a percentage. Considering that some of the items had a similar performance, and that item Q1 loaded equally on both factors, it was decided that taking this into account in the scoring provides better precision. The formula proposed is very simple, and the score can be easily calculated. The global score for the WHOQOL-AGE was computed, averaging the scores previously obtained for each factor. This is the preferred scoring method. Nonetheless, if calculating this score is not feasible, as might happen in clinical practice, it is possible to use a simpler score, obtained by adding up the items. All the results and normative values presented in the present paper have been obtained using the first scoring method, and therefore they cannot serve as a guideline if the second option is used.

In terms of reliability, Cronbach’s alpha values were higher than the recommended cut-off point of 0.70 [30], indicating adequate internal consistency. Regarding validity, the convergent validity and discriminant validity coefficients were appropriate, and the difference in the magnitude between them was sufficiently high, also supporting construct validity evidence [32]. In line with previous results for EUROHIS-QOL [4], WHOQOL-AGE also discriminates well between healthy individuals and individuals with a chronic condition, showing adequate known-groups validity. Since the results were similar across countries, only the general analyses, pooling the data of the three countries, are shown (country-by-country analyses are available from the authors upon request).

The addition of the five items provided additional explanatory variance over and above EUROHIS-QOL. Furthermore, WHOQOL-AGE showed better reliability than WHOQOL-OLD. EUROHIS-QOL does not include specific questions that are relevant for older adults, and WHOQOL-OLD has to be administered together with WHOQOL-100 or WHOQOL-BREF, which implies that none of the questionnaires was a short instrument adequate to evaluate QOL in older adults. WHOQOL-AGE is an instrument that fills this gap. By combining WHOQOL-OLD and EUROHIS-QOL, it has been possible to create WHOQOL-AGE, an instrument that evaluates specific areas of QOL that are relevant for older adults, such as satisfaction with the senses, the use of time, opportunities to achieve, intimate relationships and control, but also makes it possible to compare the QOL of the older and younger populations. Moreover, WHOQOL-AGE is short enough to be used when time is at a premium, so it is especially recommended for population-based studies that are interested in measuring QOL as an adjunct to health and functional status, as was originally considered [1], or even when further measures of well-being, social networks, and built environment are included, as in the COURAGE in Europe survey.

One of the strengths of the present study is that it uses data obtained with representative samples from three different European countries. Even though there are no strict standards for determining an acceptable response rate, the response rates found in this study can be considered adequate [37] and similar to the ones found in other general population studies recently conducted in Europe, such as SHARE (with a global response rate for the ten countries of 61.8%, ranging from 37.6% in Switzerland to 73.6% in France) [9], ELSA (individual response rate of 67%) [11] and TILDA (with a household response rate of 62%) [12].

However, there are also limitations in the present paper. Some participants were excluded from these analyses because they were not able to participate in the interview; because they finalized the interview before responding to the QOL section; or because they did not respond to some of the items. The excluded sample was therefore older than the included sample. The fact that the excluded sample had also received less years of education and was less frequently married or living with a partner could be due to the higher age in this group. Furthermore, although the percentage of participants who needed a proxy was similar in the three countries, there were more people from Spain in the excluded sample. This is due to the fact that there were more people in Spain (8.3%) that did not respond to item Q13, which asks about satisfaction with intimate relationships. Cultural differences that might make this a more sensitive question in Spain might account for the higher percentage of missing responses on this question in Spain. Nevertheless, the percentage of missing values on this question is not too high, so there is no need to consider dropping it, since it adds valuable information that is not covered by any other question. Only 4.3% of respondents who answered the QOL section did not respond to at least one question on WHOQOL-AGE. If Q13 is not considered, only 0.7% of the sample has any missing value, which suggests that the questions are easy to answer, indicating the instrument’s high feasibility. Although for validation purposes the participants who did not respond to one item were excluded, a recommendation for future studies using the WHOQOL-AGE questionnaire is to allow up to one missing value in order to compute the WHOQOL-AGE score. The scores should not be obtained if there are two or more missing values (the syntax to calculate the scores with one missing value is available upon request). Future studies might consider using a proxy instrument to evaluate QOL in older people with cognitive impairment, in order to avoid missing valuable information concerning those people with the worst health state.

Although the samples were representative of the population of the three countries, in order to avoid having small sample sizes for the oldest age groups, the “oldest old” (people 80+) were overrepresented in the sampling. Nevertheless, the normative values for subjects 90+ involved a small sample size. Another limitation is that convergent validity was assessed with a single-item question. Although the use of single-item measures has sometimes been discouraged, if the construct under study is sufficiently unidimensional, single-item measures are not necessarily inferior to multiple-item measures [38]. Moreover, the present study did not address the properties of WHOQOL-AGE in terms of sensitivity to change. Further research should also explore content validity of the WHOQOL-AGE.

## Conclusions

The WHOQOL-AGE has been shown to be a short, robust instrument that can be readily implemented in population surveys to track QOL in older adults and assess the relationship between health, QOL and their determinants, as well as to measure the impact of interventions. The instrument can also be used to compare the QOL of older and younger adults.

## Competing interests

The authors declare that they have no competing interests.

## Authors’ contributions

FFC and MM conceptualized and oversaw analyses, and wrote the article. FFC carried out the statistical analyses. MP and SC revised the statistical analysis and contributed to the interpretation of data. SC, BO, JMH, and JLAM reviewed the first draft of the manuscript. MM, SC, BTA, SK, ML, BO, JMH, and JLAM designed the study, oversaw all aspects of the study implementation, and contributed to the writing of the article. All authors made critical revision of the manuscript for important intellectual content. All listed authors participated meaningfully in the study, and they have seen and approved the final manuscript.
